# Registration in the supine position improve the accuracy of cup placement in total hip arthroplasty using a portable navigation system

**DOI:** 10.1038/s41598-023-47674-9

**Published:** 2023-11-18

**Authors:** Yohei Naito, Masahiro Hasegawa, Shine Tone, Hiroki Wakabayashi, Akihiro Sudo

**Affiliations:** Department of Orthopaedic Surgery, Mie University Graduate School of Medicine, 2-174, Edobashi, Tsu, Mie 514-8507 Japan

**Keywords:** Medical research, Outcomes research

## Abstract

Portable navigation systems have been developed for use in total hip arthroplasty (THA) in recent years. Although intraoperative registration in the lateral decubitus position or the supine position is need to create the three-dimensional coordinate system, it is not clear which position is appropriate. The purpose of this study was to assess the accuracy of cup placement in primary THA in the lateral decubitus position using an image-free handheld navigation device with registration in the lateral decubitus or the supine position, and clarify which position is appropriate. This retrospective study included 129 consecutive cementless THAs performed using an image-free handheld navigation device in the lateral decubitus position. Registration in the first 68 hips was performed in the lateral decubitus position and the last 61 hips was performed in the supine position. Postoperative cup radiographic inclination and radiographic anteversion were assessed, and the accuracy was compared between the two groups. The mean absolute errors of the postoperative measured inclination and anteversion from the target angles were 3.9° ± 2.2° and 4.8° ± 3.5° in the lateral group and 2.9° ± 2.7° and 3.2° ± 2.7° in the supine group (p < 0.05). The percentage of cups inside Lewinnek’s safe zone was 94% in the lateral group and 95% in the supine group (ns). The mean absolute values of navigation error in inclination and anteversion were 3.1° ± 2.1° and 4.2° ± 2.8° in the lateral group and 2.3° ± 2.0° and 3.1° ± 2.4° in the supine group (p < 0.05 and p < 0.05). Registration in the supine position improved the accuracy of cup insertion compared with the lateral decubitus position in THA using an image-free handheld navigation device in the lateral decubitus position.

## Introduction

Prosthetic impingement between the rim of the acetabular cup and the neck of the femoral stem is an important mechanism causing dislocation in total hip arthroplasty (THA)^1^. D'Lima et al. mentioned that optimum positioning of the components is necessary to avoid a decrease in the stable range of motion due to prosthetic impingement^2^. Lewinnek et al. reported that the dislocation rate for cup orientation with anteversion of 15 ± 10 degrees and inclination of 40 ± 10 degrees was significantly lower than that for cup orientation with outside these range^3^. Kennedy et al. revealed that a large inclination angle was a risk factor of polyethylene wear and osteolysis^4^. Although malposition of the acetabular component is recognized as a risk factor for complications, the conventional technique using the free-hand technique or a mechanically guided technique to determine intraoperative acetabular component position has resulted in inaccurate cup alignment^5^. Minoda et al. investigated acetabular component orientation in 834 primary THAs using the manual technique in the lateral decubitus position and revealed that 232 hips (27.8%) were outside the Lewinnek’s safe zone^6^. The acetabular cup placement in THA using manual technique in the lateral decubitus position seems to be inaccurate.

Among the measures to improve surgical accuracy, use of computer-assisted navigation systems such as a computed tomography (CT)-based navigation system and an image-free navigation system has been shown to help achieve optimal cup alignment^7^. However, these large-console navigation systems have the disadvantage of high cost. Furthermore, CT-based navigation systems require radiation exposure, because multidirectional fluoroscopic images must be matched with 3-dimensional (3-D) pelvic images reconstructed from preoperative CT data. The Naviswiss (Naviswiss AG, Brugg, Switzerland) is a newly introduced, image-free, handheld navigation device. The navigation unit includes an infrared stereo camera that measures the position and orientation of small tags mounted to the pelvis. The tags are mounted with bone pins to provide pelvic orientation data to the camera unit. There are two different methods of registration to create the three-dimensional coordinate system to express the positional information of the acetabular cup. One method is to register the long axis of the body in the lateral decubitus position and create the three-dimensional coordinate system combined with gravitational axis. The reference plane for cup placement is determined by the long axis of the body and gravitational axis. The other method is to register the both anterior superior iliac spines (ASISs) in the supine position and create the three-dimensional coordinate system combined with the horizontal axis. The reference plane is determined by the axis connecting bilateral ASISs and horizontal axis, and it is referred as the functional pelvic plane (FPP), in which the pelvis in the supine position is axially rotated until the bilateral ASISs are aligned in the same horizontal plane. The FPP in the supine position is often recommended as a substitute for using the anterior pelvic plane (APP) which is consisted of bilateral ASISs and the pubic tubercles, because the APP in the supine position is not always flat in a sagittal plane due to individual anterior or posterior tilt of the pelvis^8^.

The purpose of this study was to assess the accuracy of cup placement in primary THA in the lateral decubitus position using an image-free handheld navigation device with registration in the lateral decubitus or the supine position, and clarify which position is appropriate.

## Methods

From April 2020 to August 2022, 129 consecutive hips in 122 patients underwent primary cementless THA using the Naviswiss in the lateral decubitus position. There were 102 women and 20 men, with a mean age of 71 years (range 44–91 years) and a mean body mass index (BMI) of 24 kg/m^2^ (range 14–36 kg/m^2^). The preoperative diagnoses were osteoarthritis (OA) in 118 hips (Crowe group 1, 108 hips; group 2, 3 hips; group 3, 4 hips; group 4; 3 hips)^9^, osteonecrosis of the femoral head (ONFH) in 8 hips, rapidly destructive coxarthrosis (RDC) in 2 hips, and rheumatoid arthritis (RA) in 1 hip. The surgical approaches were the posterior approach in 39 hips and the superior approach in 90 hips. Both approaches were performed in the lateral decubitus position. Registration in the first 68 hips was performed in the lateral decubitus position (lateral group) and the last 61 hips was performed in the supine position (supine group). The criterion for the superior approach was slight to moderate deformity of the acetabulum or femoral head because this approach was more minimally invasive than the posterior approach. We referred to the report in which Murphy described the surgical technique of the superior approach^10^. In this study, an 8- to 10-cm incision was made starting at the tip of the greater trochanter and extending proximally, in line with the femoral shaft axis. The gluteus maximus fibers were spread, and the gluteus medius was mobilized anteriorly. The piriformis tendon was mobilized posteriorly, the gluteus minimus muscle was mobilized anteriorly, and the superior capsule was exposed. A vertical capsulotomy was performed from the trochanteric fossa to the acetabular rim. The anterior capsule and the posterior capsule were retracted, and the entire acetabulum was exposed. The subsequent procedures for excision of the labrum, reaming of the acetabulum, insertion of the cup, and repair of the capsule were similar to the posterior approach. Intraoperatively, all patients were placed in the lateral decubitus position and fixed with the conventional lateral fixation device. All patients had a SQRUM TT SHELL (Kyocera, Osaka, Japan). All surgeries in this study were performed by one experienced arthroplasty surgeon. The patients’ demographic characteristics are shown in Table 1.

**Table 1:** Patients’ demographic characteristics.

	Lateral group	Supine group	p value
Gender (women/men)	55/11	52/9	ns
Age (years)	72 (61–91)	70 (44–88)	ns
Body mass index (kg/m^2^)	25 (18–33)	24 (15–36)	ns
Diagnosis			
Osteoarthritis	61	55	ns
Crowe group 1	55	51	
Crowe group 2	1	2	
Crowe group 3	2	2	
Crowe group 4	3	0	
Osteonecrosis of the femoral head	4	4	
Rapidly destructive coxarthrosis	2	1	
Rheumatoid arthritis	1	1	
Surgical approach			
Posterior approach	23	16	ns
Superior approach	45	45	

**Figure 1:**
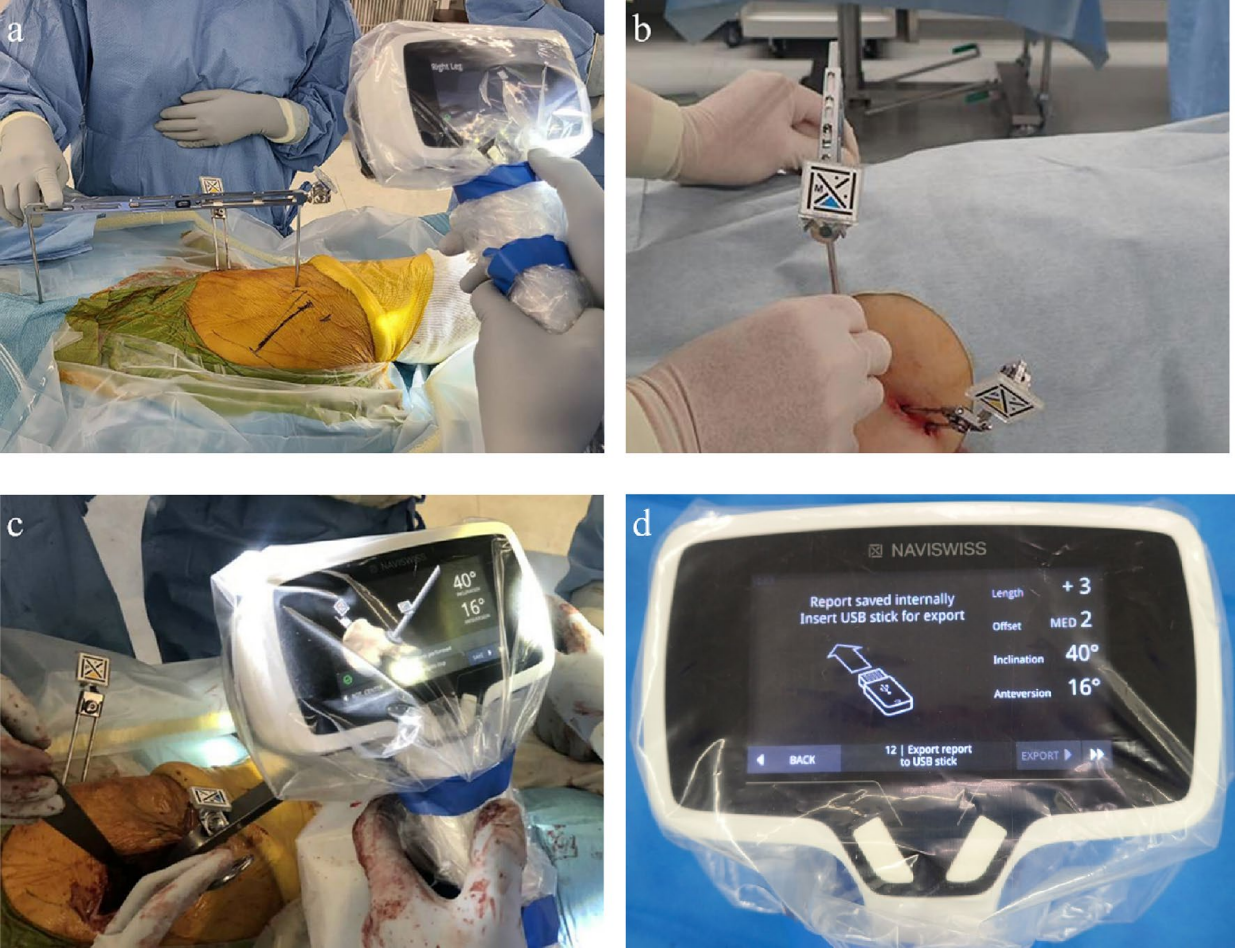
The use of the image-free handheld navigation system used in a THA surgery. Registration of the body axis using pelvic caliper in the lateral decubitus position (**a**). Registration of the both anterior superior iliac spines in the supine position (**b**). Cup impaction with reference to the values measured by the navigation device (**c**). Final cup alignment displayed on the screen (**d**).

The Naviswiss consists of a handheld navigation device and miniature precision tracking tags. Two fixation pins, 3.0 mm in diameter, were placed on the iliac crest to fix the tag (P-tag, yellow). The other tag (M-tag, blue) was attached to the pelvic caliper. In the lateral group, a point on the chest that lies on the patient’s mid chest axis, and the greater trochanter were identified. Both points were palpated with the pelvic caliper and the body axis was registered by recognizing the both P-tag and M-tag with camera (Fig. 1a). The gravity direction was registered automatically by the gravity sensor built-into the navigation unit. The gravity direction in coronal plane was the landmark for cup inclination. The plane consisted of the body axis and the gravity direction in axial plane was the landmark for cup anteversion. In the supine group, both ASISs were palpated simultaneously using pelvic caliper with M-tag and the gravity sensor automatically referenced the horizontal axis and FPP was established (Fig. 1b). After registration, patients were placed in the lateral decubitus position with management of sterility of the fixation pins. During cup impaction, M-tag was attached to the cup impactor. Cup inclination and anteversion were displayed on the screen by recognizing the both P-tag and M-tag with camera (Fig. 1c). Angles displayed on the screen were the radiographically defined angles and report was saved (Fig. 1d). The cup orientation was planned to be 40° in radiographic inclination and 15° in radiographic anteversion based on the definitions of Murray relative to the FPP^11^.

Postoperative cup position was assessed using a 3D-Template system after CT examination (ZedHip, Lexi, Tokyo, Japan). Cup radiographic inclination and anteversion were evaluated relative to the FPP by the same independent observer. The absolute values of errors of radiographic inclination and anteversion were calculated by subtracting postoperative angles from the target angles. The proportions of hips within Lewinnek’s safe zone (40° ± 10° inclination; 15° ± 10° anteversion) were analyzed. To assess the accuracy of the navigation system, absolute differences between the intraoperative values measured by the navigation device and the postoperative values measured by postoperative CT were calculated in all hips with patients’ consent. Intraoperative loosening of the fixation pins and complications such as pin-site infection, nerve injury, and dislocation were examined.

According to the previous study in which the accuracy of cup placement was compared between two types of portable navigation systems in the lateral decubitus position, the mean absolute values of navigation error in inclination were 4.6° ± 3.1° and 2.5° ± 1.7° respectively^12^. On the basis of these date, a power analysis was performed to determine sample size to detect a difference of 2.1° with a standard deviation of 2.5°. A sample size of ≥ 24 cases per group was determined to provide a power of 0.8 and two-sided α level of 0.05 using a Mann–Whitney U test. The intraclass correlation coefficient (ICC) was used to analyze intra-observer and inter-observer reliabilities. All statistical analyses were performed using SPSS version 27 software (SPSS Inc., Chicago, IL). Statistical analysis was performed using the Mann–Whitney U test for continuous variables. All continuous data was non-normally distributed. The chi-square test and Fisher’s exact test were used for categorical data. Spearman’s rank correlation coefficients were used for correlation analysis of absolute values of navigation error with age and BMI.

This study was approved by the institutional review board of Mie University hospital (H2018-083). Informed consent was obtained from all patients for the use of their surgical data. All methods were performed in accordance with the Declaration of Helsinki.

## Results

**Figure 2:**
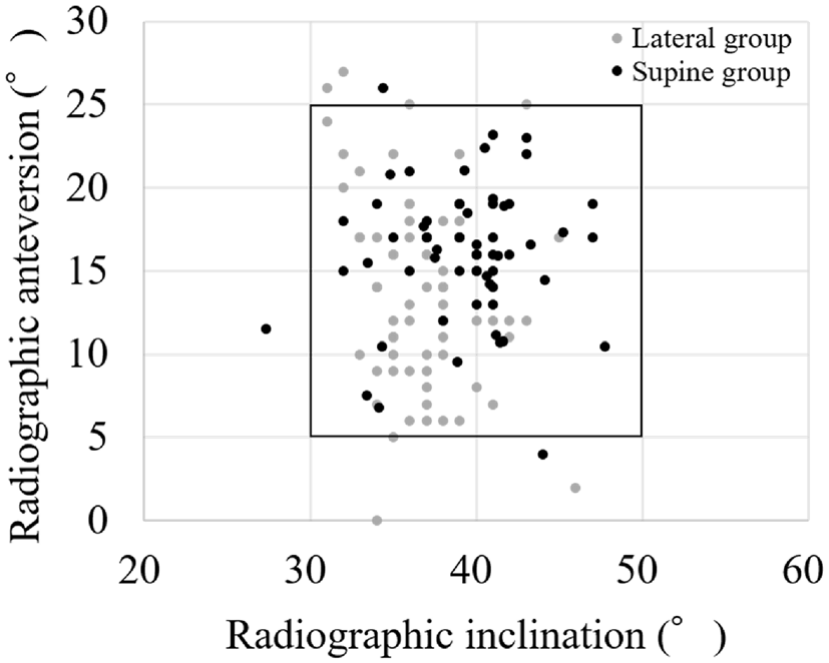
Scatterplot of the alignment of the acetabular component relative to Lewinnek’s safe zone. The percentage of cups inside the safe zone was 94% in the lateral group and 95% in the supine group (ns).

The intra-observer reliabilities were 0.973 and 0.951 for inclination and anteversion, respectively. The inter-observer reliabilities were 0.962 and 0.942 for inclination and anteversion, respectively. The mean postoperative radiographic inclinations relative to the FPP were 36.8° ± 3.2° (range 31°–46°) in the lateral group and 39.3° ± 3.9° (range 27°–48°) in the supine group. The mean postoperative radiographic anteversion relative to the FPP were 13.8° ± 5.8° (range 0°–27°) in the lateral group and 16.1° ± 4.1° (range 4°–26°) in the supine group. The mean absolute errors of the postoperative measured inclination and anteversion from the target angles were 3.9° ± 2.2° (range 0°–9°) and 4.8° ± 3.5° (range 0°–15°) in the lateral group and 2.9° ± 2.7° (range 0°–13°) and 3.2° ± 2.7° (range 0°–11°) in the supine group (p < 0.05 and p < 0.05) (Table 2). The percentage of cups inside Lewinnek’s safe zone was 94% in the lateral group and 95% in the supine group (ns) (Fig. 2). The mean absolute values of navigation error in inclination and anteversion were 3.1° ± 2.1° (range 0°–9°) and 4.2° ± 2.8° (range 0°–13°) in the lateral group and 2.3° ± 2.0° (range 0°–10°) and 3.1° ± 2.4° (range 0°–10°) in the supine group (p < 0.05 and p < 0.05) (Table 3). No significant correlations of navigation error with age and BMI were observed in either group. There were no significant differences in navigation error between women and men, OA and the others, Crowe group 1 and Crowe group 2 to 4, and posterior approach and superior approach in either group (Table 4). Intraoperative loosening of the fixation pins or complications relative to the navigation procedure were not observed in either group. No dislocations occurred in either group.

**Table 2:** Absolute values of errors of the measured postoperative angles compared to the target angles.

	Lateral group	Supine group	p value
Inclination (°)	3.9 ± 2.2	2.9 ± 2.7	< 0.05
Anteversion (°)	4.8 ± 3.5	3.2 ± 2.7	< 0.05

**Table 3:** Absolute values of navigation error.

	Lateral group	Supine group	p value
Inclination (°)	3.2 ± 2.1	2.3 ± 2.0	< 0.05
Anteversion (°)	4.2 ± 2.8	3.1 ± 2.4	< 0.05

**Table 4:** Results of statistical analysis of factors affecting navigation error.

	Lateral group		Supine group	
	Inclination	Anteversion	Inclination	Anteversion
Gender	p = 0.839	p = 0.157	p = 0.246	p = 0.478
Age	r = − 0.075, p = 0.543	r = − 0.098, p = 0.426	r = − 0.126, p = 0.331	r = 0.046, p = 0.725
Body mass index	r = − 0.247, p = 0.042	r = − 0.153, p = 0.212	r = 0.028, p = 0.831	r = 0.065, p = 0.62
Diagnosis	p = 0.59	p = 0.099	p = 0.899	p = 0.684
Crowe group	p = 0.492	p = 0.507	p = 0.266	p = 0.371
Surgical approach	p = 0.642	p = 0.434	p = 0.933	p = 0.205

## Discussion

This study demonstrated that the accuracy of cup placement using an image-free handheld hip navigation system was acceptable in primary cementless THA in the lateral decubitus position. Furthermore, registration in the supine position significantly improved the accuracy of cup placement compared with registration in the lateral decubitus position.

Saxler et al. reported that only 26% of the acetabular components positioned using the freehand technique were within the safe zone^13^. Hassan et al. reported that 58% of the components were positioned inside the safe zone despite using the mechanical alignment guide^14^. Previous studies reported that image-free large-console navigation systems improved the accuracy of cup positioning compared with the conventional free-hand technique using alignment guides. These studies reported that the percentages of cups inside the safe zone ranged from 80 to 93% with the navigation and 37% to 57% with the conventional technique^15–18^. Although image-free large-console navigation systems can be used without radiation exposure, which is the disadvantage of CT-based navigation systems, the problems of high initial costs remain. Recently, an accelerometer-based portable navigation system (HipAlign, OrthAlign, Aliso Viejo, CA), augmented reality (AR)-based portable navigation system (AR-Hip, Zimmer Biomet Japan, Tokyo, Japan), and the Naviswiss have been introduced, and studies of those accuracy have been reported^12,19–25^. The percentages of cups inside the safe zone were reported to range from 93 to 100% with the HipAlign^19–21,23^, in contrast to the percentages of 67% to 93% with the conventional technique^19,21,23^. Hasegawa et al. reported that the percentages of cups inside the safe zone was 95% with the Naviswiss and 67% with the conventional technique in the supine position^25^. The percentages of cup inside the safe zone were similar with previous reports of portable navigation systems^19,21,23,25^ (Table 5).

**Table 5:** Summary of image-free navigation systems in total hip arthroplasty.

		Position	Lewinnek’s safe zone (%)	Absolute error from target angle		Navigation error		Dislocation rate (%)
				Inclination (°)	Anteversion (°)	Inclination (°)	Anteversion (°)	
Image-free large console navigation								
VectorVision hip 3.0	Kalteis et al.^15^	Supine	93	3.6	4.2	2.9 ± 2.2	4.2 ± 3.3	0
Hiplogics Universal Protocol	Parratte et al.^16^	Supine	80	N/A	N/A	4 ± 2.8 (BMI < 27) 3.3 ± 3.06 (BMI ≥ 27)	3.4 ± 3.6 (BMI < 27) 11.6 ± 6.1 (BMI ≥ 27)	0
Navitrack	Lass et al.^17^	Supine	90	3.0 ± 2.5	5.5 ± 3.6	3.2 ± 2.4	6.5 ± 3.7	N/A
Orthopilot THApro	Takeda et al.^26^	Supine/lateral	N/A	N/A	N/A	3.7 ± 2.7	6.8 ± 3.6	0
Brainlab Hip 6.0	Naito et al.^18^	Supine/lateral	93	3.4 ± 3.0	5.1 ± 3.6	3.3 ± 2.8	5.8 ± 4.9	0.9
Portable navigation								
HipAlign	Hasegawa et al.^19^	Supine	98	3.8 ± 2.7	3.3 ± 2.5	3.7 ± 2.8	3.0 ± 2.6	0
HipAlign	Tetsunaga et al.^20^	Supine	100	N/A	N/A	3.3 ± 2.4	3.4 ± 2.2	0
HipAlign	Okamoto et al.^21^	Supine	99	3.1 ± 2.2	2.8 ± 2.3	N/A	N/A	N/A
HipAlign	Hayashi et al.^22^	Supine	N/A	2.6 ± 1.9	2.7 ± 2.2	2.7 ± 2.1	2.7 ± 1.8	0
HipAlign	Tanino et al.^23^	Lateral	93	3.7 ± 3.0	6.0 ± 4.5	N/A	N/A	0
HipAlign	Tetsunaga et al.^24^	Lateral	N/A	N/A	N/A	4.1 ± 3.7	6.8 ± 4.8	N/A
HipAlign	Tsukada et al.^12^	Lateral	N/A	N/A	N/A	4.6 ± 3.1	6.4 ± 4.2	0
AR-Hip	Tsukada et al.^12^	Lateral	N/A	N/A	N/A	2.5 ± 1.7	2.1 ± 1.8	0
Naviswiss	Hasegawa et al.^25^	Supine	95	4.1 ± 3.2	4.3 ± 3.2	2.8 ± 2.2	2.8 ± 2.0	0
Naviswiss	Present study	Lateral	94^a^	3.9 ± 2.2^a^	4.8 ± 3.5^a^	3.0 ± 2.1^a^	4.3 ± 2.9^a^	0^a^
			95^b^	2.9 ± 2.7^b^	3.2 ± 2.7^b^	2.3 ± 2.0^b^	3.1 ± 2.4^b^	0^b^

^a^Registration in lateral decubitus position, ^b^Registration in supine position.

Previous studies reported that the navigation error of image-free large-console navigation systems ranged from 2.9° to 3.7° in inclination and 4.2° to 6.8° in anteversion^15,17,18,26^. In the previous studies of the HipAlign, the navigation error was reported to range from 2.7° to 4.6° in inclination and 2.7°–6.8° in anteversion^12,19,20,22,24^. Tsukada et al. reported that the navigation error of the AR-Hip was 2.5° in inclination and 2.1° in anteversion^12^. Hasegawa et al. reported that the navigation error of the Naviswiss in the supine position was 2.8° in inclination and 2.8° in anteversion^25^. The navigation error in the present study was comparable to previous studies of portable navigation systems^12,19,20,22,24,25^ (Table 5). There was no correlation between navigation error and BMI in this study, whereas Hasart et al. reported that BMI and soft tissue thickness affected the accuracy of cup orientation in THA using image-free navigation system^27^. Because all patients were Japanese in this study, the mean BMI was relatively lower than that in American or European patients. There were no cases with BMI categorized into class II (BMI ≥ 35 kg/m^2^) or class III (BMI ≥ 40 kg/m^2^) based on the World Health Organization definitions in the lateral group, and only one case was categorized into class II and no cases were categorized into class III in the supine group^28^. Relatively low BMI might have affected the results of correlation analysis between navigation error and BMI. Several reports from Japan using potable navigation systems also reported that no correlation was observed between navigation error and BMI^19,21,25^. Furthermore, there were no correlations between navigation error and diagnosis or Crowe group. The deformity might not affect the navigation error because there was no need to register the acetabulum. Surgical approach had no effect on navigation error in the present study. The reason might be that registration was completed before incision.

Previous studies reported that the dislocation rate of THA using image-free large-console navigation systems ranged from 0 to 0.9%, in contrast to ranging from 0% to 1.5% using the conventional technique^15,16,18,26^. The dislocation rate of THA using the HipAlign was reported to be 0%, in contrast to ranging from 0 to 1.8% with the conventional technique^12,19,20,22,23^. Hasegawa et al. reported that no dislocations occurred in any hips using the Naviswiss or the conventional technique in the supine position^25^. No dislocation was occurred in this study (Table 5).

The cup orientation was planned to be 40° in radiographic inclination and 15° in radiographic anteversion relative to the FPP in the supine position in all cases in this study. Recent studies mentioned that the pelvic tilt in standing and sitting position should be regarded as a reference of acetabular cup in the preoperative planning because the degree of acetabular cup anteversion was directly related to the degree of pelvic tilt with the change of body position^29,30^. Hepinstall et al. reported that considering preoperative pelvic tilt in standing position based on standing preoperative radiographs may improve the postoperative standing functional cup placement in primary THA^31^. Ramkumar et al. demonstrated the usefulness of a patient specific safe zone mathematically derived from the change in pelvic tilt in sitting and standing position for cup placement in revision THA due to instability^32^. Because almost all cups were inside the target zone in this study, it may be possible to place the cups inside the patient specific safe zone with the Naviswiss taking into account the preoperative pelvic tilt in standing position and/or sitting position, however, further study is necessary to prove it.

Registration in the supine position provided more precise cup placement compared with registration in the lateral decubitus position. Previous studies revealed that progressive changes of pelvic tilt angles from the supine position occurred after placing the patients in the lateral decubitus position. Kanazawa et al. reported that the absolute values of change in pelvic tilt in the supine position and the lateral decubitus position were 5.0° ± 4.1° in the sagittal plane, 5.2° ± 4.0° in the axial plane, and 2.8° ± 2.3° in the coronal plane^33^. Iwakiri et al. reported that the mean sagittal tilt of the pelvis relative to the FPP was − 5.0° ± 4.8° (backward tilt) when fixed in the lateral decubitus position with the conventional lateral position fixation device^34^. In the lateral group, inaccurate patient positioning during registration in the lateral decubitus position seemed to be one of the reasons of less accurate cup placement. Furthermore, although the preoperative planning and the postoperative measurement were conducted relative to the FPP, the cups were placed relative to the plane consisted of the body axis and the gravity direction which was different from the FPP. The difference of these planes also should have resulted in the inaccuracy of cup placement. On the other hand, in the supine group, because the plane consisted of the axis connecting both ASISs and the horizontal axis which were registered in the supine position was the FPP, it was possible to place the acetabular cups relative to the FPP. Furthermore, because the patients were placed in the lateral decubitus position after the FPP was acquired, inaccurate patient positioning in the lateral decubitus position did not affect the accuracy of cup placement. These should have been the reasons of improvement of the accuracy of cup placement with registration in the supine position.

To our best knowledge, there were no reports to compare the accuracy of cup placement between the registration in the lateral decubitus position and the supine position using the same navigation system. Tsukada et al. reported that the AR-Hip allowed the surgeon to create the three-dimensional coordinate system with the patient in the supine position and enabled the surgeon to achieve more precise cup placement compared with the HipAlign which created the three-dimensional coordinate system with the patient in the lateral decubitus position^12^. Although different types of navigation system were used, the study was similar to our study in terms of comparing the registration in the lateral decubitus position and the supine position, and a power analysis was performed on the basis of these previous data.

This study has some limitations. First, it included no comparison with conventional free-hand arthroplasty. However, the accuracy of cup orientation in the present study was comparable to that of previous studies using image-free navigation systems. The results of the present study suggested the usefulness of the Naviswiss to improve the accuracy of cup insertion compared to free-hand technique. Second, the clinical outcomes were not investigated. Long-term follow-up is necessary to show the clinical advantages to use the Naviswiss.

In conclusion, the accuracy of cup placement using an image-free handheld hip navigation system was acceptable in primary cementless THA in the lateral decubitus position. Registration in the supine position significantly improved the accuracy of cup placement compared with registration in the lateral decubitus position.

## Data Availability

The datasets during and/or analyzed during the current study are available from the corresponding author on reasonable request.
